# A case report of severe calciphylaxis – suggested approach for diagnosis and treatment

**DOI:** 10.1186/s12882-017-0556-z

**Published:** 2017-04-21

**Authors:** Margret Patecki, Gabriele Lehmann, Jan Hinrich Bräsen, Jessica Schmitz, Anna Bertram, Lars Daniel Berthold, Hermann Haller, Wilfried Gwinner

**Affiliations:** 1 Department of Nephrology and Hypertension, Hannover Medical School, Carl-Neuberg-Straße 1, 30635 Hannover, Germany; 2 Division of Rheumatology/Osteology, Department of Internal Medicine III, Friedrich-Schiller-University of Jena, Erlanger Allee 101, 07747 Jena, Germany; 3 Institute of Pathology, Hannover Medical School, Carl-Neuberg-Straße 1, 30635 Hannover, Germany; 4 Institute for Diagnostic and Interventional Radiology, Hannover Medical School, Carl-Neuberg-Straße 1, 30635 Hannover, Germany

**Keywords:** Case report, Mineral metabolism and bone disease, CKD complications, Calciphylaxis, Bone biopsy, Adynamic bone disease

## Abstract

**Background:**

Calciphylaxis is a serious complication in patients with chronic kidney disease associated mineral and bone disorder. It can occur in conditions with low and high bone turnover. So far, there are no definite diagnostic and therapeutic guidelines which may prevent the devastating outcome in many calciphylaxis patients. We report a case which clearly illustrates that knowledge of the underlying bone disorder is essential for a directed treatment. Based on this experience we discuss a systematic diagnostic and therapeutic approach in patients with calciphylaxis.

**Case presentation:**

We report a patient with severe calciphylaxis. Initial evaluation showed an elevated serum parathormone concentration and a bone-specific alkaline phosphatase activity in the upper normal range; however, the bone biopsy clearly showed adynamic bone disease. Extended dialysis with low calcium dialysate concentration and citrate anticoagulation, and administration of teriparatide led to a further increase in bone-specific alkaline phosphatase activity and most importantly, resulted in an activated bone turnover as confirmed by a second bone biopsy 11 weeks later.

**Conclusions:**

This case illustrates that laboratory tests cannot reliably differentiate between high and low bone turnover in calciphylaxis patients. More importantly, this case highlights the fact that specific therapies that alter bone metabolism cannot be applied without knowledge of the bone status. On this background, we suggest that bone biopsies should be an integral part in the diagnosis and therapeutic decision in these patients and should be evaluated in further studies.

## Background

Calciphylaxis is a life-threatening complication in patients with kidney disease. It is also called “calcific uremic arteriolopathy”, reflecting the main findings of medial calcification and intimal hyperplasia in skin biopsies of calciphylaxis patients. The lesions are usually very painful and ulcerations and necrosis can develop [1]. Besides the involvement of skin, visceral and muscle arteries can be affected. Complications, particularly septic episodes are common and explain the high mortality rate of about 45–80% [2]. The diagnosis is based on the clinical features in combination with known risk factors, i.e. renal insufficiency, obesity, female gender, hyperparathyroidism and disturbed bone metabolism, therapy with vitamin K antagonists, inflammatory states, and diabetes mellitus [2, 3]. A skin biopsy can prove presence of calciphylaxis; however, it is usually dispensable and even can set a new nidus for calciphylaxis [4]. Imaging studies can strengthen the diagnosis of calciphylaxis and help to assess the extent of muscular and inner organ involvement [5, 6]. The pathophysiology of calciphylaxis is largely unknown. A disturbed calcium-phosphate homeostasis in association with an altered bone metabolism (either high or low turnover) is probably one of the important factors [7–9].

Current treatment recommendations rely on weak evidence and therapy failures are common [8]. Supportive wound management, antibiotic therapy, and analgesics are certainly an important part of the therapy [10]. Cessation of calcium supplements and medication that increases serum calcium levels such as thiazide diuretics and active vitamin D compounds is generally recommended [2, 8, 11]. Avoiding vitamin K antagonists or even supplementation of vitamin K has been suggested based on the role of vitamin K in the γ-carboxylation of proteins that regulate local calcium crystal deposition [12]. The broad array of suggested treatment options is probably a reflection of the uncertain efficacy: sodium thiosulfate, bisphosphonates, cinacalcet, emergency parathyroidectomy, plasma exchange and optimization of the dialysis treatment [2, 8, 11, 13]. Moreover, some of these therapies are powerful effectors of the bone metabolism, particularly bisphosphonates, cinacalcet, and parathyroidectomy which can drastically reduce bone turnover [14, 15]. For thiosulfate, no direct effects on bone turnover have been reported so far [16]. With our case, we want to highlight an important, but so far less considered aspect for an individual treatment strategy: the assessment of the underlying bone metabolism by a bone biopsy to adjust the treatment strategy accordingly.

## Case presentation

A 54-years old obese female patient was hospitalized in July 2015 with severe cutaneous calciphylaxis. She suffered from congenital cystic kidney disease and had been undergoing hemodialysis since 1996. She received her first kidney transplant in 1999, which failed in 2004 because of acute rejection. A second allograft transplanted in 2009 had a complicated course with acute T cell- and antibody-mediated rejections and despite treatment, development of chronic antibody-mediated rejection. Allograft function never exceeded a GFR (cystatin C) of 27 mL/min. First symptoms of cutaneous calciphylaxis began after failure of the first allograft. At this time, intact parathormone (iPTH) level was 510 pg/mL, serum calcium 2.25 mmol/L, serum phosphate 1.71 mmol/L, and alkaline phosphatase activity 69 units/L. Gluteal regions, lower limbs, abdomen, and mammae were affected, requiring surgery and skin transplantation. Bone pain or fractures had never occurred. Subtotal parathyroidectomy in 2006 and total parathyroidectomy in 2009 were not successful to control hyperparathyroidism. Therefore, cinacalcet was administered from 2009 until March 2015. The iPTH ranged between 265 and 463 pg/mL, the alkaline phosphatase activity between 61 and 110 units/L, and serum calcium between 2.0 and 2.3 mmol/L during this treatment. Nevertheless, smouldering calciphylaxis symptoms remained.

**Fig. 1:**
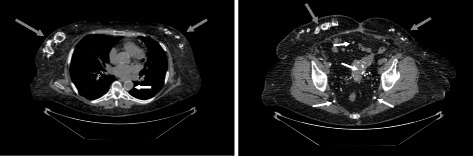
Extraosseous calcifications detected by CT imaging. *Left*: Unenhanced thoracic computed tomography with severe subcutaneous calcifications of both mammae, pronounced on the right side (*grey arrows*), bronchial calcifications and nearly circular aortosclerosis (*white arrow*). *Right*: Precontrast abdominal computed tomography with disseminated subcutaneous calcifications in the abdominal wall (*grey arrows*) and in smaller pelvic arteries (*white arrows*)

**Fig. 2:**
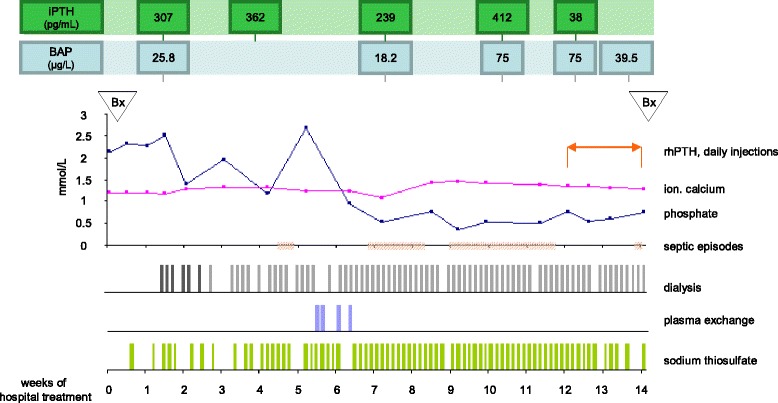
Laboratory results and treatment. Time course of ionized serum calcium (*pink line*; normal range: 1.14–1.27 mmol/L) and serum phosphate (*blue line*; normal range: 0.83–1.67 mmol/L) during the 14 weeks of hospital treatment. Septic episodes are indicated on the abscissa (*light red*). ‘Bx‘indicates the time point of bone biopsy and ‘rhPTH, daily injections‘the duration of teriparatide treatment (20 μg per day). Intact parathormone (iPTH; *dark green boxes*; normal range: 10–65 pg/mL, suggested range for CKD 5D: 2–9 times the upper normal range [21]) and bone alkaline phosphatase (BAP; *light blue boxes*; normal range: 5–27 μg/L) are shown in the upper part at the corresponding time points. Dialysis frequency and sodium thiosulfate administration (25 g per infusion) are shown in the bottom part of the graph (*dark grey lines* indicate use of a dialysate calcium of 1.25 mmol/L and heparin anticoagulation, light grey lines a dialysate calcium of 1.0 mmol/L and citrate anticoagulation)

At admission, the medication included tacrolimus, mycophenolate mofetil, prednisolone, cholecalciferol, alphacalcidol, calcium acetate, sevelamer, a thiazide diuretic, three antihypertensive drugs, bicarbonate, pantoprazole, simvastatin, and insulin for type II diabetes which had been present since 2012. Coumadin had been given for deep vein thrombosis, but was discontinued 3 months prior admission. Body weight was 96.6 kg at a height of 162 cm. Painful cutaneous lesions were present at multiple sites of the lower trunk, not restricted to the locations of insulin injection. CT imaging additionally showed severe calcifications of the abdominal arteries (Fig. 1). Initial laboratory evaluation indicated inflammation, with a C-reactive protein of 259 mg/L (normal < 9 mg/L) and leukocytosis of 13.5/nL (normal range 3.9–10.2/nL). Hemoglobin was 7.7 g/dL (normal ≥ 12.0 g/dL). Serum protein was 52 g/L (normal ≥ 65 g/L). Renal function was marginal but unchanged to previous values (serum creatinine 374 μmol/L, cystatin C eGFR 11 mL/min). The cholecalciferol level was 4.5 ng/mL (normal 20–50 ng/mL). Initial and subsequent levels of ionized calcium, phosphate, iPTH, and bone-specific alkaline phosphatase activity (BAP) as well as subsequent specific treatments are shown in Fig. 2.

At first, calcium-containing drugs, vitamin D compounds, and the thiazide were discontinued. A low-calcium diet and vitamin K supplementation were prescribed. Infusion of sodium thiosulfate was begun, and because of worsening metabolic acidosis during this treatment, hemodialysis was initiated. To achieve better control of calcium and phosphate levels and to intensify sodium thiosulfate therapy, dialysis sessions were extended (≥6 h, 5–7 times/week). Furthermore, four plasma exchanges were performed with fresh frozen plasma according to recent suggestions [13]. Supportive therapy included antibiotic prophylaxis with clindamycin, opioide analgesics and interdisciplinary wound care. The course was further complicated by recurrent septic episodes of unknown origin and an atraumatic bowel perforation, a rare complication in systemic calciphylaxis [17].

**Fig. 3:**
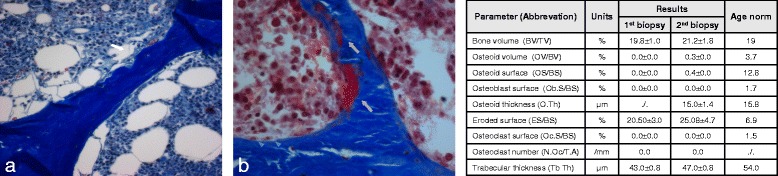
Bone histology from the iliac crest at admission (**a**) and after treatment with intensified dialysis and rhPTH (**b**). **a** Missing osseous remodeling and cellular paucity is present. Empty resorption lacunae [*white arrow*] indicate former osteoclast activity. Masson-Goldner stain (magnification × 200). **b** The trabecular surface is covered with lining cells (*blue arrow heads*), intratrabecular and endosteal osteoid layers (*grey arrows*), indicative of an activated bone turnover. Masson-Goldner stain (magnification × 400). Standard histomorphometric parameters of both biopsies are shown in the table

To evaluate the bone metabolism after prolonged cinacalcet treatment, a bone biopsy (without prior tetracycline labelling) had been performed shortly after admission. When the histology results were obtained and showed adynamic bone disease (Fig. 3a), the treatment was adapted to stimulate bone metabolism by inducing a negative calcium balance. For this, dialysate calcium was reduced to 1.0 mmol/L, citrate anticoagulation was used, and extended daily dialyses sessions were maintained. After 7 weeks, increased iPTH and BAP suggested an activated bone metabolism. Dependence on daily dialysis and temporary dialysis access problems led us to explore an additional therapeutic option, recombinant human PTH (teriparatide). Teriparatide induces bone turnover [18] and is indicated for low turnover osteoporosis [19, 20]. No increase in serum calcium was observed during daily treatment with 20 μg teriparatide and the patient reported improvement of pain. Notably, the duration of dialysis sessions could be reduced to 4 hours without impairment of calcium levels. Unfortunately, the patient died from septic shock a few weeks later. We obtained permission to take a postmortem bone biopsy. The biopsy revealed resorption lacunae at multiple sites, several lining cells and osteoid deposition, indicative of an activated bone turnover (Fig. 3b).

## Discussion and Conclusions

We present a patient with a complex history of calciphylaxis. The underlying bone disease was certainly the result of long term end stage renal failure and of two poorly functioning kidney allografts. Hyperparathyroidism had been present for more than 10 years and despite cinacalcet therapy and achieving calcium, phosphate, and iPTH serum levels within the recommended ranges [21], the patient continuously had flares of calciphylaxis.

Our patient had several predisposing factors for calciphylaxis [2], such as hyperparathyroidism, obesity, and diabetes that had been present for a long time. Nevertheless, the immediate trigger for the acute worsening of calciphylaxis is unknown: There was no sudden drop in renal allograft function as GFR was consistently below 20 mL/min after an anti-rejection therapy 3 months prior to the crisis. Moreover, coumadin therapy had been stopped several months before and dosage of vitamin D and calcium-based phosphate binders had not been changed recently.

Initial therapeutic interventions were in accordance with the general recommendations [2, 8, 11], including normalization of serum calcium and phosphate levels by dialysis, cessation of calcium-containing phosphate binders, vitamin D, and thiazide diuretics, substitution of vitamin K, and sodium thiosulfate treatment dose-adjusted to the renal function. The patient also received a trial of plasmapheresis, without clinical improvement.

According to the current guidelines [21] a bone biopsy should be considered in conditions with unclear mineral and bone disturbances, e.g. fractures or unexplained hypercalcemia. We decided to take a bone biopsy shortly after admission for several reasons: (*i*) calciphylaxis may occur in both, high and low turnover bone disease [9], (*ii*) our patient had a prolonged therapy with cinacalcet which can suppress bone turnover [15], and (*iii*) we were uncertain whether laboratory parameters such as BAP and iPTH are sufficiently informative in this situation.

The initial histomorphometric analysis revealed low turnover osteopathy, an unexpected result in view of the moderately high iPTH and a BAP in the upper normal range. Consequently, daily dialysis with low calcium and citrate anticoagulation was initiated to induce hypocalcemia and hypophosphatemia, with the aim to minimize extraosseous calcium crystal deposition and to stimulate bone turnover by hypocalcemia-triggered PTH synthesis. After 7 weeks of this intensified treatment an increased iPTH and BAP was observed.

Our subsequent trial with teriparide was well tolerated, resulted in a reduction of pain, and was not associated with increases in serum calcium despite reduced dialysis intensity. At the end, bone turnover was significantly activated as illustrated by a sustained increase of BAP and the histologic findings in the post-mortem bone biopsy. It is certainly difficult to conclude which of the treatments (intensified dialysis or teriparatide) was the major contributor to the improved bone metabolism. Increases in iPTH and BAP were already observed with intensified dialysis alone. It remains speculative whether bone activation by teriparatide helped to maintain stable calcium levels. Because of the short observation time after initiation of teriparatide we were unable to evaluate the long-term evolution of BAP under this treatment. Also, we could not determine whether alternative treatment schedules (i.e. once per week; [22]) would have been more beneficial. It should be noted that occurrence of calciphylaxis has been reported during teriparatide treatment [23]; yet it is unknown if reduced or increased bone turnover was present in these patients. Of course, high turnover bone disease would be a contraindication for teriparatide. Also, reliable control of serum calcium and awareness of potential deterioration of calciphylaxis is mandatory in any patient receiving this treatment.

Similar to observations from Sugimto et al., iPTH levels dropped shortly after teriparatide administration [24]. This probably reflects a negative feedback of endogenous PTH generation in response to teriparatide administration. Teriparatide (rhPTH 1–34) in turn, is not detected by the iPTH laboratory test. We can only speculate why an endogenous PTH level of greater than 300 pg/mL at admission was associated with adynamic bone metabolism and in turn, why application of teriparatide should be efficient in this situation. First, endogenous PTH can be oxidized in uremia which is associated with decreased biological activity [25]. Further, administration of teriparatide leads to a considerable increase in 1–34 PTH levels up to 10fold above the upper normal physiologic PTH concentrations [26].

Our case demonstrates that identification of the underlying bone metabolism is of crucial importance for an appropriate and specific treatment of calciphylaxis. Laboratory results cannot sufficiently reflect the status of bone turnover which has been reported before [27]. In our case, initial iPTH and BAP analyses were suggestive of normal or high turnover but the bone biopsy clearly showed adynamic bone disease. Therefore, we believe that in most cases with calciphylaxis a bone biopsy should be considered to avoid erroneous and potentially harmful treatments. For example, in our patient parathyroidectomy, treatment with bisphosphonates or cinacalcet would have aggravated adynamic bone disease and thus, probably impaired the calciphylaxis.

**Fig. 4:**
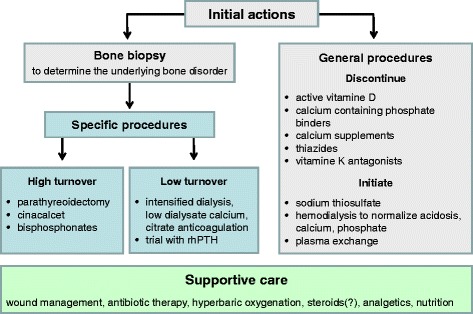
Suggested procedures for patients with calciphylaxis. This flow chart shows a possible treatment path for calciphylaxis patients which has to be evaluated in further studies. In addition to the general recommended interventions, a bone biopsy should be performed initially to determine the underlying real bone disease (*grey boxes*). Once a diagnosis of high or low bone turnover has been established specific procedures (*blue boxes*) can be initiated. Supportive care (*green box*) should be provided at any time. Recommendations for general procedures, high turnover bone disease and supportive care were adopted from [2, 8, 11]

Our suggestion therefore is a gradual approach that should be evaluated in further studies (Fig. 4). Initially, general actions should include stopping of medication that can precipitate hypercalcemia, normalization of high serum calcium and phosphate levels, administration of sodium thiosulfate [2, 8, 11, 13]. Plasma exchange might be considered but procedural risks including infections should be balanced against potential benefits. As early as possible, a bone biopsy should be considered to determine the type of bone disorder. Ideally, assessment of bone metabolism includes tetracycline labelling. However, the labelling procedure delays early diagnosis and directed treatment and thus, should be decided individually. Once the bone disorder is characterized bone metabolism can be corrected – either by reducing or increasing bone turnover – to prevent further deterioration of calciphylaxis.

## Abbreviations

(e) GFR: (estimated) glomerular filtration rate
BAP: Bone-specific alkaline phosphatase activity
iPTH: Intact parathormone
rhPTH: Recombinant human parathormone
